# Past Year Cannabis Use Among Norwegian Adolescents: Time Trends Based on the Ungdata Surveys 2010–2019

**DOI:** 10.3389/fpsyt.2021.627479

**Published:** 2021-03-16

**Authors:** Ove Heradstveit, Sondre Aasen Nilsen, Kyrre Breivik, Anders Bakken, Thomas Haug, Kristian Hartveit, Kjell Morten Stormark

**Affiliations:** ^1^Regional Centre for Child and Youth Mental Health and Child Welfare, NORCE Norwegian Research Centre, Bergen, Norway; ^2^Center for Alcohol & Drug Research, Stavanger University Hospital, Stavanger, Norway; ^3^Norwegian Social Research (NOVA), OsloMet–Oslo Metropolitan University, Oslo, Norway; ^4^KoRus Vest, Bergen Haukeland University Hospital, Bergen, Norway; ^5^Department of Health Promotion and Development, Faculty of Psychology, University of Bergen, Bergen, Norway

**Keywords:** cannabis use, trends, adolescents, population-based survey, substance use, Ungdata

## Abstract

**Aims:** To describe trends in cannabis use from 2010 to 2019 among Norwegian adolescents and relate these to individual- and municipal-level variables.

**Design:** Data from nationwide repeated cross-sectional surveys collected in 2010–2013 (T1), 2014–2016 (T2), and 2017–2019 (T3) were used to describe secular trends in proportions of adolescent cannabis use.

**Setting:** Cross-sectional surveys in 410 of the total 428 municipalities of Norway.

**Participants:** A total of 628,678 survey responses from adolescents aged ~13–19 years of age, in which 566,912 survey responses were eligible for analyses, representing data from 340 municipalities.

**Measurements:** Respondent's past year cannabis use, time, gender, school grade, municipality, geographical location, and municipality population.

**Findings:** Boys reported overall higher cannabis use, with ~2:1 gender ratio for any past year cannabis use and a 3:1 gender ratio for frequent cannabis use. Adolescents in Eastern Norway reported higher cannabis use compared with other areas in the country, and adolescents from municipalities with a higher population size reported higher rates of cannabis use than smaller municipalities. A gradual increase in cannabis use from T1 to T3 was found in Eastern Norway and in the largest municipalities. More generally, proportions of past year cannabis use showed a marked increase from T2 to T3 across genders, grade/age groups, geographical location, and municipality population, with few exceptions.

**Conclusions:** Our findings indicate that proportions of past year cannabis use have increased among Norwegian adolescents in recent years. Preventive interventions to hinder initiation of cannabis use, as well as measures to address frequent cannabis use among Norwegian adolescents, are needed.

## Introduction

Cannabis is the most widely used illicit drug globally (1) with relatively high prevalence rates among adolescents and young adults in several Western countries (2–5). Cannabis use is associated with mental health problems, failing to attend classes, difficulties with concentration, and reductions in motivation (6–11). Adolescents who use cannabis appear to be more vulnerable to potential adverse effects compared with adults (12). Measuring trends in cannabis use are complex and are affected by a number of individual- and community-level variables that are important to consider to understand ongoing changes.

As patterns of use may change, epidemiological studies on time trends are important to monitor the extent and correlates of cannabis use across different settings. There are mixed findings on time trends in current use of cannabis. In some regions (e.g., western Europe, USA, and Australia), cannabis use appears to have stabilized or declined, after a period of increased use throughout the 1990s and early 2000s (1). A study based on the Health Behavior in School-aged Children (HBSC) study, which included 160,606 adolescents aged 15 years from 30 European and North American countries, reported that the frequency of lifetime cannabis use generally decreased from 2002 to 2010 in Europe and North America (13). The decrease occurred after a prolonged period of normalization, whereby the normative acceptance of using these drugs in the adolescent group had now been reduced (13, 14).

On the other hand, findings suggest that cannabis use increased in other countries characterized by a low baseline prevalence, such as Austria, Macedonia, and Latvia—however, only for boys (13). This may suggest a normalization of cannabis use in these areas. As Norway traditionally has had a fairly low adolescent cannabis use compared with many other European countries (15), one could argue that normalization of cannabis use has yet to become evident here. Results from the European School Survey Project on Alcohol and Other Drugs (ESPAD) survey showed that cannabis use among Norwegian 15- to 16-year-olds peaked around 2000, whereby 12% reported past year use, before declining to 6% in the period from 2007 to 2015 (15, 16). Importantly, a marked increase in cannabis use was again observed in the period from 2015 to 2019, with ~9% of the adolescents reporting cannabis use (16). Recent results from the nationally representative Ungdata survey also suggest that cannabis use is on the rise (17), and a significant increase in illicit drug use among Norwegian higher education students from 2014 to 2018 has been reported (18). However, it is unclear whether the general increase of cannabis use indicated by the Ungdata report is generalizable across age groups, genders, and geographical locations within Norway. A more extensive investigation of these data is needed in order to shed light on time trends in cannabis use among Norwegian adolescents.

Across geographical areas and time, adolescent boys tend to report higher cannabis use in general and more often develop cannabis abuse and dependence than girls (19). While a closing gender gap has been observed during the past decades in relation to cannabis use (20), data from a more recent time period suggest an opposite tendency in many Western countries (13). Changes in cannabis use may also differ across subregions of a country, for example, across urban and rural areas (21).

### The Present Study

The present study is based on data from a set of large, cross-sectional surveys conducted among adolescents in Norway yearly since 2010 (“Ungdata”). This provides a unique opportunity to study time trends in cannabis use among Norwegian adolescents. Our study sought to:

1. Describe time trends in cannabis use over the period from 2010 to 2019 in a large range of municipalities of Norway among adolescents in eighth to tenth grade elementary school and first to third grade upper secondary school (roughly corresponding to 13–19 years of age).
2. Examine how gender, age groups, municipality, geographical location, and municipality population relate to time trends in cannabis use.

## Materials and Methods

### Study Population and Procedures

Ungdata is a cross-national data collection scheme designed to conduct surveys of adolescents in Norway at the municipality level (for more information, see www.ungdata.no/english/). Ungdata is regarded as the most comprehensive source of information on adolescent health, lifestyle, and well-being in Norway. The municipalities themselves order the Ungdata survey, and around 150 questions are similar across all surveys, while additional questions are available to be chosen by each municipality that participates. Ungdata surveys are mainly conducted from January to May, and the questionnaires are completed at school. The vast majority of Norwegian adolescents attend elementary school, while school non-attendance is somewhat higher at upper secondary school level. Specifically, ~95% of all 16–18-year olds in Norway are enrolled in upper secondary education (22). In the earliest years of the Ungdata survey, relatively few municipalities participated and primarily on the elementary school level. From 2013 to 2019, a majority of the municipalities of Norway have participated, many on several occasions, and the upper secondary school level has been gradually more represented. The surveys are financed by the Norwegian Directorate of Health, and participation is therefore free of charge for participating municipalities. Ungdata surveys target adolescents in secondary schools (grades 8–10; ~13–16 years of age) and upper secondary schools (first to third year; ~16–19 years of age) in Norway. The Ungdata surveys are administered by NOVA (Norwegian Social Research) in cooperation with regional drug and alcohol competence centers (KoRus).

We used data from a large set of Ungdata surveys that has been conducted in almost all Norwegian municipalities in the period from 2010 to 2019. Because most municipalities performed one Ungdata survey every 3 years, the dataset was split into 3 time periods: T1 (2010–2013), T2 (2014–2016), and T3 (2017–2019). The dataset from each period was representative at both the national and regional levels. The total number of survey responses in our data was 628,678 (49.9% girls). The response rates were 82% for elementary school and 63% for upper secondary school in both T1 and T2 and 87% for elementary school and 73% for upper secondary school in T3.

The study followed the Declaration of Helsinki guidelines. Collection of data from elementary school-age adolescents was anonymous and did not need approval by data protection agencies. Collection of data from upper secondary school-age adolescents was approved by the Norwegian data protection authority. The manuscript does not contain clinical studies or patient data.

### Instruments

#### Cannabis Use

In the present study, the outcome variable was self-reported past year cannabis use. The item read “How many times during the past 12 months have you tried hashish or marijuana?” and response categories included “never,” “once,” “2–5 times,” “6–10 times,” and “11 times or more.” We used these levels unchanged in an ordinal variable for total past year cannabis use. In addition, we dichotomized the variable to separate between those reporting no past year cannabis use (coded “0”) from those reporting one or more times with any past year cannabis use (coded “1”). Finally, we constructed a variable for frequent past year cannabis use that separated those with “11 times or more” of past year cannabis use (coded “1”) from those with no or less past year cannabis use (coded “0”).

#### Sociodemographic Variables

The respondents were asked to indicate their gender and school grade (i.e., a marker of age group). School grade included the last 3 years of Norwegian secondary school (i.e., eighth, ninth, and tenth grade, corresponding to age groups of 13–14, 14–15, and 15–16 years of age by the time of the survey), as well as the first 3 years of upper secondary school (i.e., first, second, and third year, corresponding to age groups of 16–17, 17–18, and 18–19 years of age by the time of the survey).

#### Municipal-Level Variables

##### Municipality

Data on each of the respondent's municipality were available in the dataset. Specifically, this refers to the municipality of the school that the adolescent attends. For elementary school-age adolescents, the municipality of the school is primarily the same as their municipality of living. For upper secondary school-age adolescents, the municipality of the school may not be the same as the municipality of living, as many adolescents attend school in a different (often neighboring) municipality. To be able to compare municipalities that were rearranged during the time period from 2010 to 2019, these municipalities were recoded into the new organization of the municipalities of interest. This recoding was done for three municipalities that were recoded into one municipality (*n* = 4,787).

##### Geographical location

We divided all participants into their municipalities' geographical location based on the county code that was available through the municipality variable (see Appendix 1 in Supplementary Material for details). The following values were assigned to the geographical location variable: “Eastern Norway,” “Southern Norway,” “Western Norway,” “Central Norway,” and “Northern Norway.” According to national statistics, 82% of the Norwegian population live in urban settlements, with the highest density of inhabitants living in urban vs. rural settlements in Oslo (99.4%) and the lowest in Oppland (59.3%) (23).

##### Municipality population

One variable for municipality population was constructed using official data from Statistics Norway on population sizes in each of the municipalities of Norway per 2019 (24). In the case that no population size was available per 2019, we used the latest available statistics on population size. We coded this variable into the five population size ranges (25): “Below 5,000,” “5,000 to 9,999,” “10,000 to 19,999,” “20,000 to 49,999,” and “above 50,000.” Survey responses with invalid municipality codes were coded as missing in this variable (see Appendix 1 in Supplementary Material for details).

### Missing Data

We excluded all survey responses with either missing data on gender, age groups/school grade, and/or cannabis use from all analyses (*n* = 61,766). Thus, 566,912 survey responses were eligible for our analyses, representing 90.2% of the available survey responses in the Ungdata dataset. The excluded survey responses were significantly different from the included survey responses on gender, age, geographical location, municipality size, and cannabis use (all ps < 0.001). Effect sizes for these differences were, however, small (Cohen's *d* ranging from 0.17 to 0.20), except for a moderate effect size for municipality size (*d* = 0.43) and a very small effect size for cannabis use (*d* = 0.04). See Appendix 2 in Supplementary Material for details on distribution of missing responses in our sample. Also, survey responses with missing data on the variable of municipality size (*n* = 1,604; 0.3% of total) was omitted from analyses where the municipality size variable was part of the specific analysis.

### Statistical Analysis

The following statistical analyses were conducted. First, to assess the overall characteristics of the Ungdata survey, we described the number of Norwegian municipalities that have participated in the survey, as well as rates of repeated participation in the survey. Second, to describe the sample, we provided descriptive characteristics of gender, age groups, time periods, geographical location, municipality population, and participation across survey years, while missing data on relevant variables were also described. Third, to analyze trends in cannabis use, proportions of total past year cannabis use were calculated across time periods for each gender and adjusted for age group, geographical location, and municipality size. Similarly, proportions of any and frequent past year cannabis use were calculated across survey time points (T1–T3) for each age group stratified by gender and adjusted for geographical location and municipality size. Also, proportions of any and frequent past year cannabis use were calculated by geographical location and municipality size adjusted for age group and gender. Finally, to control for sampling characteristics, we also performed sensitivity analyses investigating rates of any and frequent past year cannabis use across time periods, where we compared the total sample with subsamples consisting of municipalities that had participated in every time period (i.e., in T1, T2, and T3).

All proportions were calculated using a multiple logistic regression model for associations between time periods and cannabis use, after which the “margins” command in STATA was used to calculate rates of cannabis use per survey year (26). As we were interested in adjusted rates, we added relevant control variables in these analyses (i.e., gender, age groups, geographical location, and municipality population) and were thus able to establish adjusted rates of cannabis use with 95% confidence intervals. Statistical significance of change was reported separately for each time period compared with each previous time period using an alpha of *p* < 0.01 to determine a significant increase/decrease. Trend plots were visualized using the R-package “Ggplot2.” All other analyses were performed using STATA version 16 (27).

## Results

### The Sample

A total of 61,766 survey responses were excluded from the analyses due to missing gender (*n* = 17,442), missing age group (*n* = 20,063), missing cannabis use (*n* = 20,174), or missing on several of these variables (*n* = 4,087). The excluded subjects were more often boys (59.6 vs. 49.4% in the full sample) and comprised relatively more adolescents from higher age groups and municipalities with a small population compared with the included individuals (Appendix 2 in Supplementary Material). Among the excluded individuals, 5.5% reported any past year cannabis use compared with 6.6% among the included individuals.

As shown in Table 1, ~96% of the municipalities of Norway (410 out of 428) were registered with any participation in the Ungdata surveys from T1 to T3. In the included sample, ~80% of the municipalities of Norway were represented. Of these, 99.0% of the survey responses were from municipalities that had participated at least twice in the Ungdata surveys. In the included sample (*n* = 566,912), 55.0% (*n* = 311,774) had participated in all time periods (i.e., T1, T2, and T3; see Table 1 for details).

**Table 1:** Frequency of participation by unique municipalities in Ungdata surveys.

	**Total sample**, ***N*** **=** **628,678**		**Included sample**, ***N*** **=** **566,912**		
	**Survey responses *N* (%)**	**Municipalities *N* (%)^**a**^**	**Survey responses *N* (%)**	**Municipalities *N* (%)^**a**^**	**Participation in T1, T2, and T3 *N***
Once	7,109 (1.1)	41 (9.6)	5,834 (1.0)	26 (6.1)	0
Twice	122,640 (19.6)	151 (35.3)	109,497 (19.4)	116 (27.1)	0
Three times	332,908 (53.1)	151 (35.3)	303,523 (53.7)	133 (31.1)	176,579^d^
Four times	153,512 (24.5)	64 (15.0)	136,543 (24.1)	62 (14.5)	125,284
Five times	10,687 (1.7)	3 (0.7)	9,911 (1.8)	3 (0.7)	9,911
Total	626,856 (100.0)^b^	410 (95.8)	565,308 (100.0)^c^	340 (79.4)	311,774^e^

a *The percentage is relative to the total number of municipalities in Norway (i.e., n = 428)*.

b *Omitting eight municipality codes that only included county code and could therefore not be identified as a specific municipality, representing n = 1,822 of the survey responses (0.3% of total). See Appendix 1 in Supplementary Material*.

c *Omitting seven municipality codes that only included county code and could therefore not be identified as a specific municipality, representing n = 1,604 of the survey responses (0.3% of total)*.

d *These survey responses (n = 176,579) are used in sensitivity analyses as subsample II (Figure 7)*.

e *These survey responses (n = 311,774) are used in sensitivity analyses as subsample I (Figure 7)*.

As outlined in Table 2, the number of survey responses rose from 121,767 at T1 (representing 175 unique municipalities) to 272,268 at T3 (representing 310 unique municipalities). The gender composition was fairly equal across the time periods. While the earliest surveys had relatively few respondents from second and third year at upper secondary school (i.e., 17–19-year olds), the composition of age group evened out during the most recent time period. Adolescents from Eastern Norway comprised ~half of the eligible responses in the Ungdata surveys. Composition of municipality size was fairly equal across T1–T3.

**Table 2:** Descriptive characteristics of the included individuals (*n* = 566,912).

	**Total**	**T1**	**T2**	**T3**
	**2010–2019**	**2010–2013**	**2014–2016**	**2017–2019**
	***N* = 566,912*n* (%)**	***N* = 121,767*n* (%)**	***N* = 172,877*n* (%)**	***N* = 272,268*n* (%)**
**Gender**				
Boys	280,243 (49.4)	60,016 (49.3)	85,502 (49.5)	134,725 (49.5)
Girls	286,669 (50.6)	61,751 (50.7)	87,375 (50.5)	137,543 (50.5)
**Grade**				
Elementary Eighth	119,574 (21.1)	29,641 (24.3)	36,784 (21.3)	53,149 (19.5)
Elementary Ninth	116,658 (20.6)	28,767 (23.6)	36,580 (21.2)	51,311 (18.8)
Elementary Tenth	117,813 (20.8)	31,568 (25.9)	36,229 (21.0)	50,016 (18.4)
Upper First	103,679 (18.3)	20,621 (16.9)	32,003 (18.5)	51,055 (18.8)
Upper Second	69,432 (12.3)	8,189 (6.7)	20,612 (11.9)	40,631 (14.9)
Upper Third	39,756 (7.0)	2,981 (2.4)	10,669 (6.2)	26,106 (9.6)
**School level**				
Elementary school-age	354,045 (62.4)	89,976 (73.9)	109,593 (63.4)	154,476 (56.7)
Upper secondary school-age	212,867 (37.6)	31,791 (26.1)	63,284 (36.6)	117,792 (43.3)
**Geographical location**				
Eastern Norway	281,995 (49.7)	48,464 (39.8)	90,039 (52.1)	143,492 (52.7)
Western Norway	146,245 (25.8)	42,733 (35.1)	36,640 (21.2)	66,872 (24.6)
Northern Norway	52,354 (9.2)	9,557 (7.8)	17,480 (10.1)	25,317 (9.3)
Central Norway	46,244 (8.2)	14,518 (11.9)	12,047 (7.0)	19,679 (7.2)
Southern Norway	40,074 (7.1)	6,495 (5.3)	16,671 (9.6)	16,908 (6.2)
**Municipality size**				
<5,000	36,790 (6.5)	6,356 (5.2)	14,175 (8.3)	16,259 (6.0)
5,000–9,999	69,268 (12.2)	15,227 (12.5)	23,073 (13.4)	30,968 (11.4)
10,000–19,999	105,023 (18.6)	25,905 (21.3)	32,078 (18.7)	47,040 (17.3)
20,000–49,999	143,968 (25.5)	30,401 (25.0)	41,477 (24.2)	72,090 (26.5)
50,000+	210,259 (37.2)	43,811 (36.0)	60,809 (35.4)	105,639 (38.8)
*Unknown^a^*	*1,604*	*67*	*1,265*	*272*
**Unique municipalities**	340 (79.4)^b^	175 (40.9)^b^	271 (63.3)^b^	310 (72.4)^*b*^
**Repeated participation in T1, T2, and/or T3**^**c**^				
Once	9,496 (1.7)	55 (0.1)	957 (0.6)	8,484 (3.1)
Twice	244,038 (43.2)	34,689 (28.5)	69,561 (40.5)	139,788 (51.4)
All	311,774 (55.2)	86,956 (71.5)	101,094 (58.9)	123,724 (45.5)

*Once = only T1, only T2, or only T3. Twice = T1 + T2, T1 + T3, or T2 + T3. All = T1 + T2 + T3*.

a *Due to municipality code that only included county code and which therefore could not be identified as a specific municipality (total: n = 1,604)*.

b *Percentage is relative to the total number of municipalities in Norway (n = 428)*.

c *Repeated participation refers to specific participation in T1, T2, and T3*.

### Trends of Cannabis Use

#### Trends in Total Cannabis Use by Gender and Age Group

The results presented in Table 3 show that no change in rates of past year cannabis use was observed from T1 to T2, while rates of cannabis use increased significantly for both boys and girls from T2 to T3 (all ps < 0.001). Also, boys had higher proportions of past year cannabis use than girls, and this difference was most pronounced by T3 for both any cannabis use (gender ratio 1.7:1) and frequent cannabis use (gender ratio 3.2:1).

**Table 3:** Trends in proportions of past year cannabis use (*n* = 566,912).

	**T1 2010–2013**	**T2 2014–2016**	**T3 2017–2019**
	**% (95% CI)**	**% (95% CI)**	**% (95% CI)**
**Boys**			
None	93.1 (92.8, 93.3)	93.0 (92.8, 93.2)	90.5 (90.3, 90.6)
- Once	2.5 (2.4, 2.7)	2.4 (2.3, 2.5)	3.0 (2.9, 3.1)
−2–5 times	2.1 (1.9, 2.2)	2.0 (1.9, 2.1)	2.8 (2.7, 2.9)
−6–10 times	0.7 (0.6, 0.7)	0.7 (0.6, 0.7)	0.9 (0.9, 1.0)
−11 times or more	1.7 (1.6, 1.8)	1.9 (1.8, 2.0)	2.8 (2.7, 2.9)
Change^c^	n/a	– ^a^	>T1, T2^b^
**Girls**			
None	95.8 (95.6, 96.0)	95.6 (95.5, 95.7)	94.5 (94.4, 94.7)
- Once	1.8 (1.7, 1.9)	1.9 (1.8, 2.0)	2.3 (2.2, 2.3)
−2–5 times	1.4 (1.3, 1.6)	1.4 (1.3, 1.5)	1.8 (1.8, 1.9)
−6–10 times	0.4 (0.3, 0.4)	0.4 (0.4, 0.5)	0.5 (0.5, 0.5)
−11 times or more	0.6 (0.5, 0.7)	0.7 (0.6, 0.7)	0.9 (0.8, 0.9)
Change^c^	n/a	–^a^	>T1, T2^b^
**Gender ratio (boys:girls)**			
Any use	1.6:1	1.6:1	1.7:1
Frequent use	2.7:1	2.8:1	3.2:1

*The estimates are adjusted for age, geographical location, and municipality population*.

*Individuals with missing data on municipality population (n = 1,604) are excluded from the analysis*.

a *Compared with proportions in T1*.

b *Compared with proportions in T1 and T2*.

c *Changes at p value < 0.001 are specified in the table: “>” implies significant increase; “ < ” implies significant decrease; and “–” implies no change compared with previous time period(s)*.

**Figure 1:**
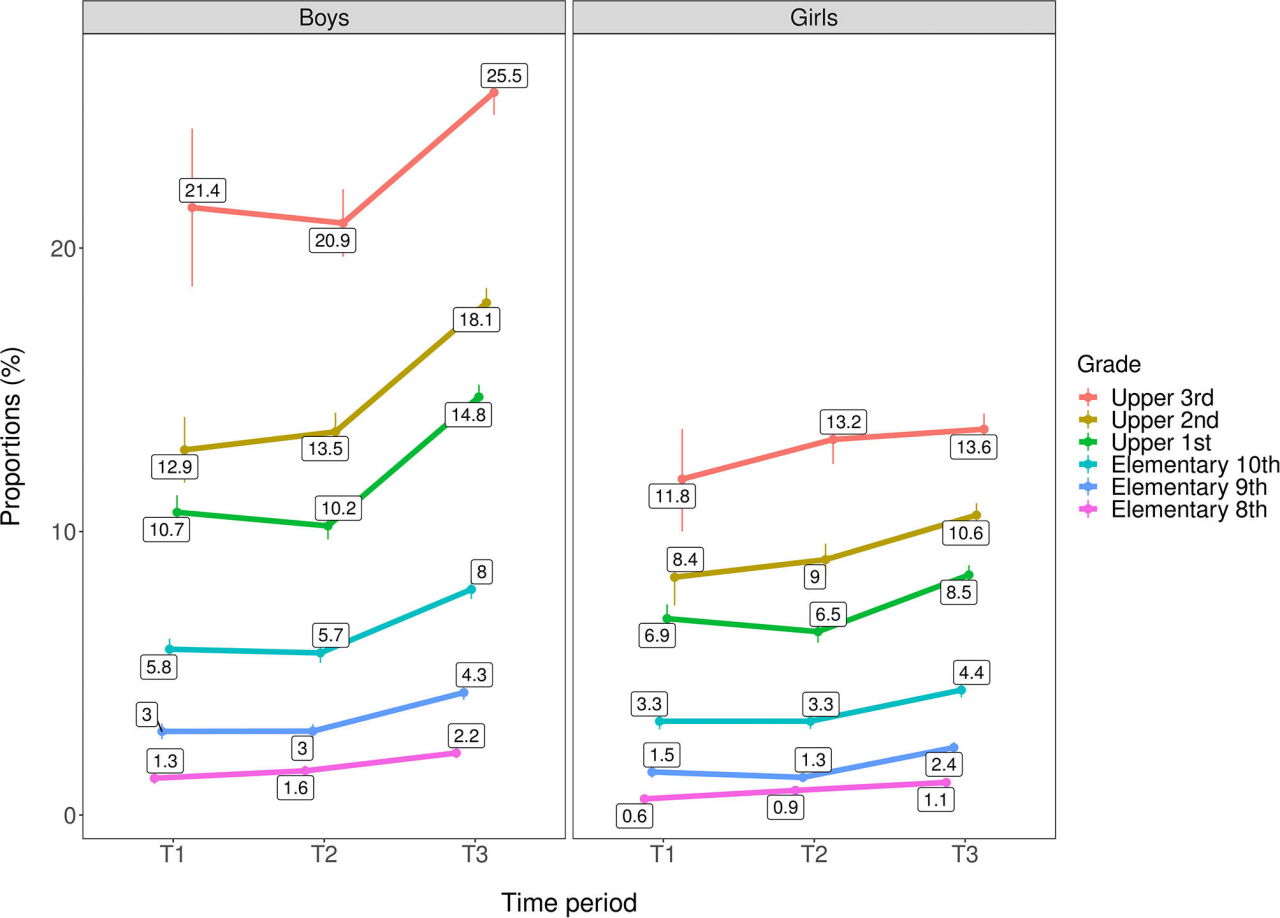
Trends in any past year cannabis use by age group/grade across genders (*n* = 566,912). T1 = 2010–2013. T2 = 2014–2016. T3 = 2017–2019. Rates are adjusted for geographical location and municipality population. Individuals with missing data on municipality population (*n* = 1,604) are excluded from the analysis.

**Figure 2:**
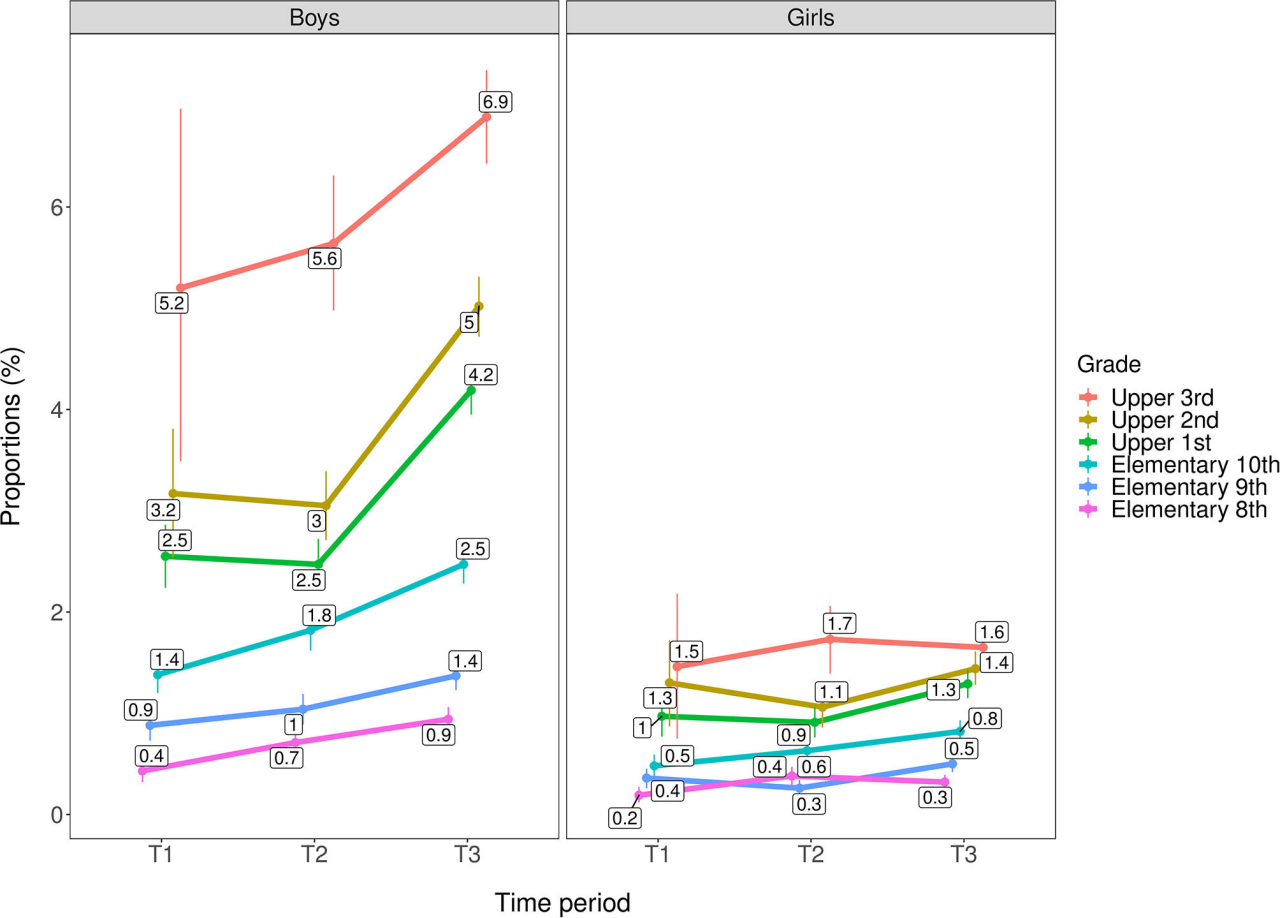
Trends in frequent past year cannabis use by age group/grade across genders (*n* = 566,912). T1 = 2010–2013. T2 = 2014–2016. T3 = 2017–2019. Rates are adjusted for geographical location and municipality population. Individuals with missing data on municipality population (*n* = 1,604) are excluded from the analysis.

As shown in Figures 1, 2 the most robust change in cannabis use was seen from T2 to T3, where the rates of any and frequent cannabis use increased across age groups for both genders. An important exception was that no change was observed for girls in third upper secondary school. Also, any cannabis use gradually increased from T1 to T3 for eighth grade elementary school-age girls only, and frequent cannabis use gradually increased from T1 to T3 for eighth and tenth grade elementary school-age boys only. All changes described above refer to ps ≤ 0.01. Generally, rates of any and frequent past year cannabis use were relatively higher among adolescents with higher age for both genders. The highest proportion of past year cannabis use was among youth in third grade upper secondary school (i.e., 18–19-year olds), with a 1.9:1 gender ratio for any past year cannabis use (boys: 25.5%; girls: 13.6%) and 4.3:1 for frequent past year cannabis use (boys: 6.9%, girls: 1.6%). For details, see Table 4.

**Table 4:** Trends in past year cannabis use by gender/age (n = 566,912).

	**Any**					**Frequent**				
	**(T1)**	**(T2)**		**(T3)**		**(T1)**	**(T2)**		**(T3)**	
	**Prop (95% CI)**	**Prop (95% CI)**	**Change^**a**^**	**Prop (95% CI)**	**Change^**a**^**	**Prop (95% CI)**	**Prop (95% CI)**	**Change^**a**^**	**Prop (95% CI)**	**Change^**a**^**
**Boys**										
Eighth Elementary	1.3 (1.1, 1.5)	1.6 (1.4, 1.7)	–	2.2 (2.0, 2.4)	>T1, T2	0.4 (0.3, 0.5)	0.7 (0.6, 0.8)	> T1*	0.9 (0.8, 1.1)	>T1, T2*
Ninth Elementary	3.0 (2.7, 3.2)	3.0 (2.7, 3.2)	–	4.3 (4.1, 4.6)	>T1, T2	0.9 (0.7, 1.0)	1.0 (0.9, 1.2)	–	1.4 (1.2, 1.5)	>T1, T2*
Tenth Elementary	5.8 (5.5, 6.2)	5.7 (5.4, 6.1)	–	8.0 (7.6, 8.3)	>T1, T2	1.4 (1.2, 1.6)	1.8 (1.6, 2.0)	> T1*	2.5 (2.3, 2.7)	> T1, T2
First Upper	10.7 (10.1, 11.3)	10.2 (9.7, 10.7)	–	14.8 (14.3, 15.2)	> T1, T2	2.5 (2.2, 2.9)	2.5 (2.2, 2.7)	–	4.2 (4.0, 4.4)	> T1, T2
Second Upper	12.9 (11.7, 14.0)	13.5 (12.9, 14.2)	–	18.1 (17.5, 18.6)	> T1, T2	3.2 (2.6, 3.8)	3.0 (2.7, 3.4)	–	5.0 (4.7, 5.3)	> T1, T2
Third Upper	21.4 (18.7, 24.2)	20.9 (19.7, 22.1)	–	25.5 (24.7, 26.3)	> T1*, T2	5.2 (3.5, 7.0)	5.6 (5.0, 6.3)	–	6.9 (6.4, 7.4)	> T2*
**Girls**										
Eighth Elementary	0.6 (0.5, 0.7)	0.9 (0.7, 1.0)	> T1*	1.1 (1.0, 1.3)	> T1, T2*	0.2 (0.1, 0.3)	0.4 (0.3, 0.5)	> T1*	0.3 (0.3, 0.4)	–
Ninth Elementary	1.5 (1.3, 1.7)	1.3 (1.2, 1.5)	–	2.4 (2.2, 2.6)	> T1, T2	0.4 (0.3, 0.5)	0.3 (0.2, 0.3)	–	0.5 (0.4, 0.6)	> T2
Tenth Elementary	3.3 (3.0, 3.6)	3.3 (3.0, 3.6)	–	4.4 (4.2, 4.7)	> T1, T2	0.5 (0.4, 0.6)	0.6 (0.5, 0.8)	–	0.8 (0.7, 0.9)	> T1
First Upper	6.9 (6.4, 7.4)	6.5 (6.1, 6.9)	–	8.5 (8.1, 8.8)	> T1, T2	1.0 (0.8, 1.2)	0.9 (0.8, 1.1)	–	1.3 (1.2, 1.4)	> T1*, T2*
Second Upper	8.4 (7.4, 9.4)	9.0 (8.5, 9.6)	–	10.6 (10.2, 11.0)	> T1, T2	1.3 (0.9, 1.7)	1.1 (0.9, 1.3)	–	1.4 (1.3, 1.6)	> T2*
Third Upper	11.8 (10.1, 13.6)	13.2 (12.4, 14.1)	–	13.6 (13.1, 14.2)	–	1.5 (0.8, 2.2)	1.7 (1.4, 2.1)	–	1.6 (1.4, 1.9)	–

*T1 = 2010/2013; T2 = 2014/2016; T3 = 2017/2019. Prop, proportions; CI, confidence interval; Any, any past year cannabis use; Frequent, frequent past year cannabis use*.

a *“>” implies significant increase; “ < ” implies significant decrease; and “–” implies no change compared with previous time period(s)*.

*All changes p < 0.001 unless specified by asterisk: p ≤ 0.01*.

#### Trends in Cannabis Use by Geographical Location

**Figure 3:**
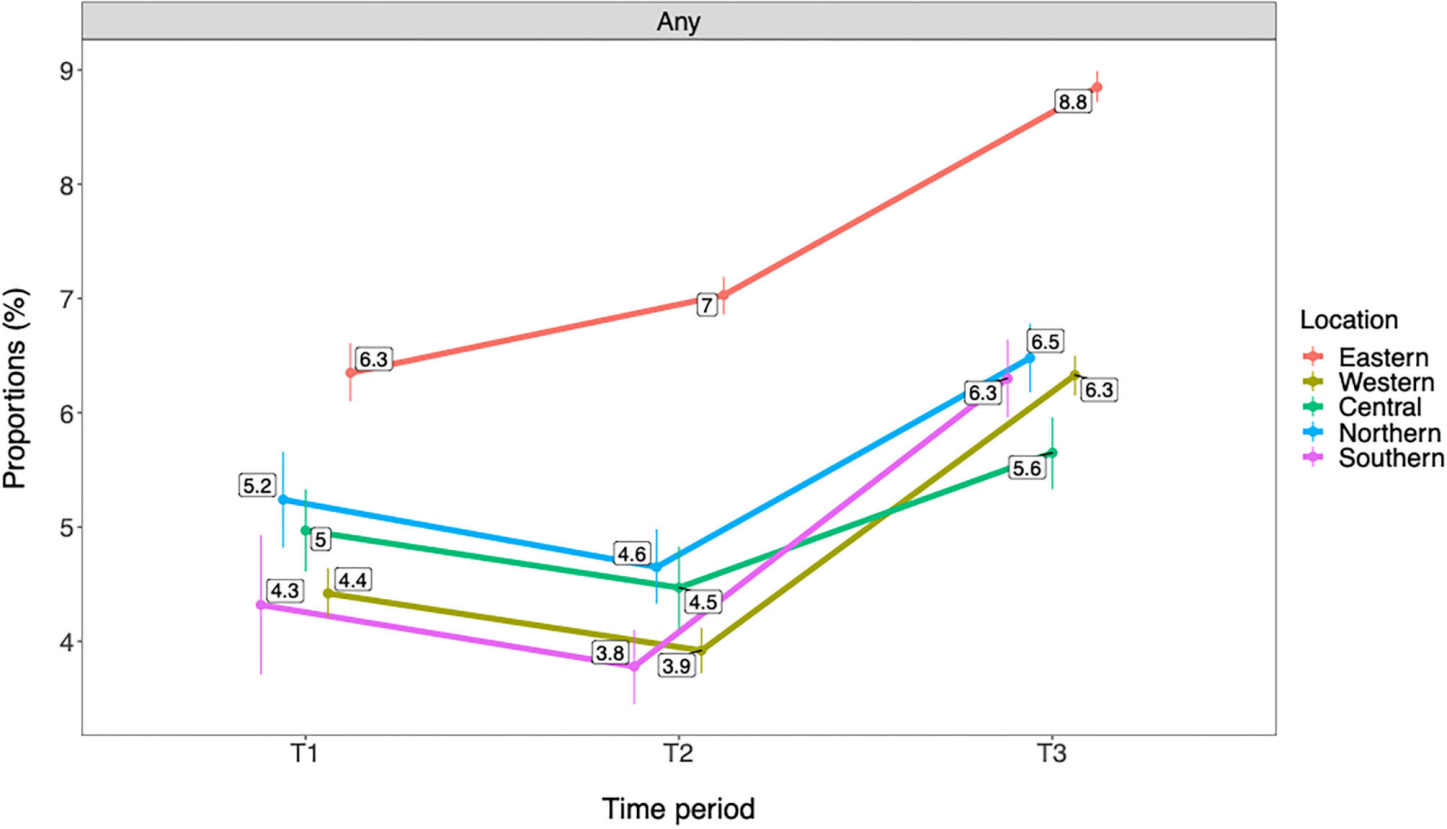
Trends in any past year cannabis use by geographical location (*n* = 566,912). T1 = 2010–2013. T2 = 2014–2016. T3 = 2017–2019. Rates are adjusted for gender and age group/grade. Any = any past year cannabis use.

**Figure 4:**
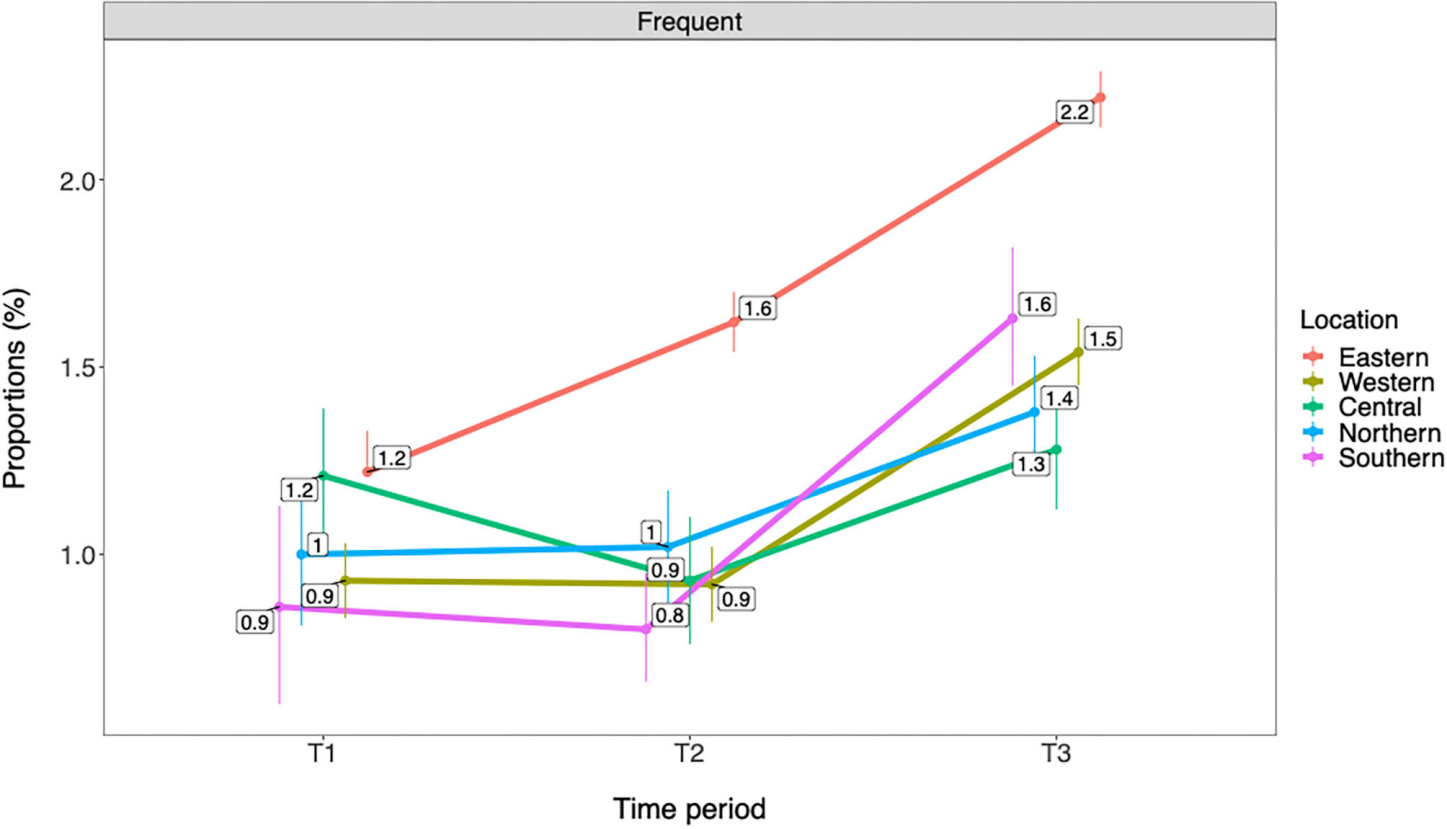
Trends in frequent past year cannabis use by geographical location (*n* = 566,912). T1 = 2010–2013. T2 = 2014–2016. T3 = 2017–2019. Rates are adjusted for gender and age group/grade. Frequent = frequent past year cannabis use.

As shown in Figures 3, 4 adolescents from Eastern Norway had consistently higher rates of any and frequent past year cannabis use compared with other geographical locations across all time periods. At T3, rates in Eastern Norway were 8.8% for any cannabis use and 2.2% for frequent cannabis use compared with 5.6–6.5% in other regions for any use and 1.3 to 1.6% for frequent use. In Eastern Norway, rates for both any and frequent cannabis use gradually increased from T1 to T3. A decreased rate from T1 to T2 was observed for Western Norway for any cannabis use. For all geographical areas, rates for both any and frequent cannabis use increased from T2 to T3. All changes described above refer to ps ≤ 0.01. See Table 5 for details.

**Table 5:** Trends in any and frequent past year cannabis use by geographical location (*n* = 566,912).

	**(T1)**	**(T2)**		**(T3)**	
	**Prop (95% CI)**	**Prop (95% CI)**	**Change**	**Prop (95% CI)**	**Change**
**Any use**					
Eastern Norway	6.3 (6.1, 6.6)	7.0 (6.9, 7.2)	> T1	8.8 (8.7, 9.0)	> T1, T2
Southern Norway	4.3 (3.7, 4.9)	3.8 (3.5, 4.1)	–	6.3 (6.0, 6.6)	> T1, T2
Western Norway	4.4 (4.2, 4.6)	3.9 (3.7, 4.1)	< T1*	6.3 (6.2, 6.5)	> T1, T2
Central Norway	5.0 (4.6, 5.3)	4.5 (4.1, 4.8)	–	5.6 (5.3, 6.0)	> T1, T2
Northern Norway	5.2 (4.8, 5.7)	4.6 (4.3, 5.0)	–	6.5 (6.2, 6.8)	> T1, T2
**Frequent use**					
Eastern Norway	1.2 (1.2, 1.3)	1.6 (1.5, 1.7)	> T1	2.2 (2.1, 2.3)	> T1, T2
Southern Norway	0.9 (0.6, 1.1)	0.8 (0.7, 1.0)	–	1.6 (1.5, 1.8)	> T1, T2
Western Norway	0.9 (0.8, 1.0)	0.9 (0.8, 1.0)	–	1.5 (1.5, 1.6)	> T1, T2
Central Norway	1.2 (1.0, 1.4)	0.9 (0.8, 1.0)	–	1.3 (1.1, 1.4)	> T2*
Northern Norway	1.0 (0.8, 1.2)	1.0 (0.9, 1.2)	–	1.4 (1.2, 1.5)	> T1*, T2*

*T1 = 2010/2013; T2 = 2014/2016; T3 = 2017/2019. Prop, proportions; CI, confidence interval*.

*“>” implies significant increase; “ < ” implies significant decrease; and “–” implies no change compared with previous time period(s)*.

All changes p < 0.001 unless specified by asterisk: p ≤ 0.01

#### Trends in Cannabis Use by Municipality Population

**Figure 5:**
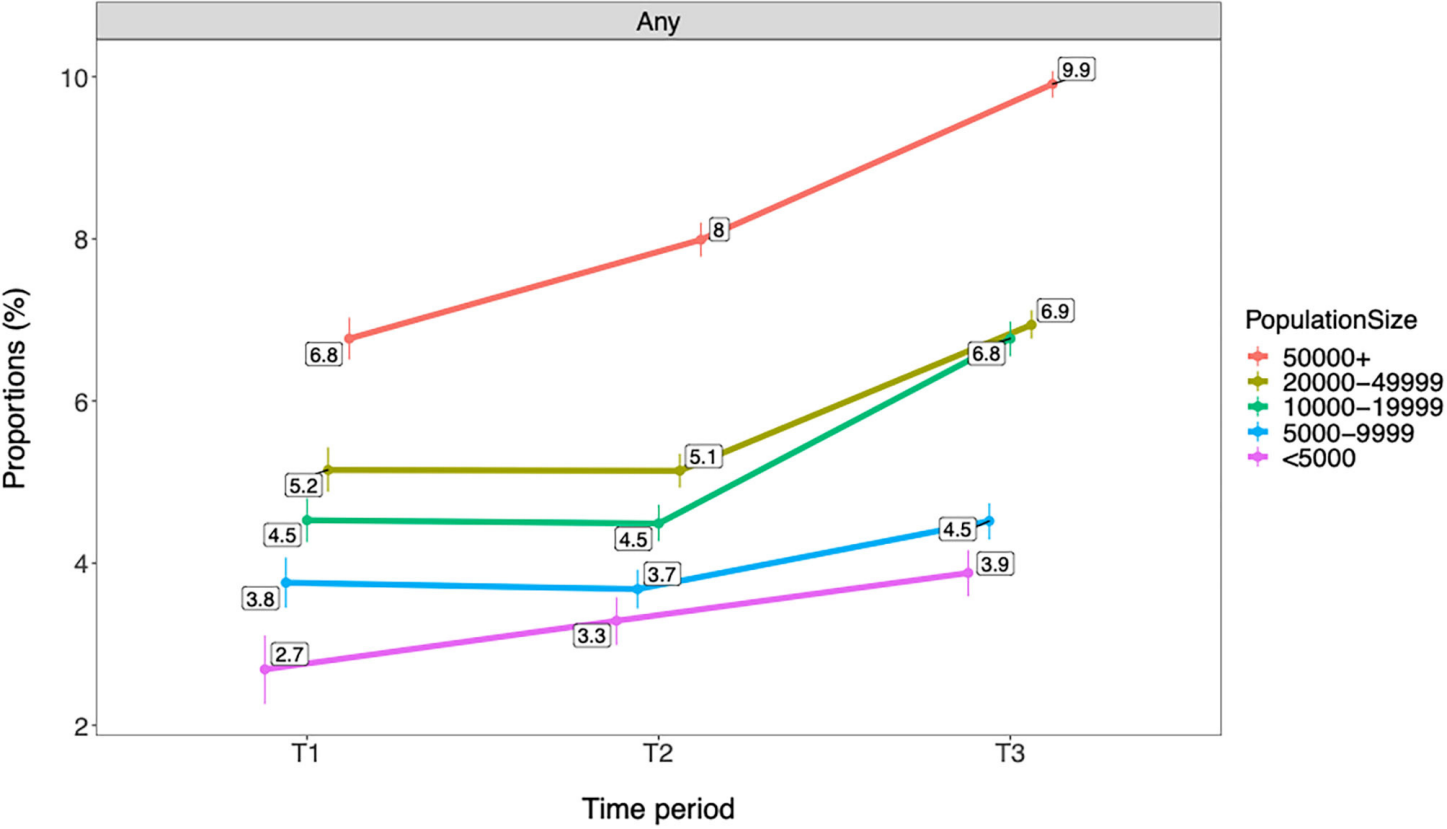
Trends in any past year cannabis use by municipality population (*n* = 566,912). T1 = 2010–2013. T2 = 2014–2016. T3 = 2017–2019. Proportions are adjusted for gender and age group/grade. Individuals with missing data on municipality population (*n* = 1,604) are excluded from the analysis. Any = any past year cannabis use.

**Figure 6:**
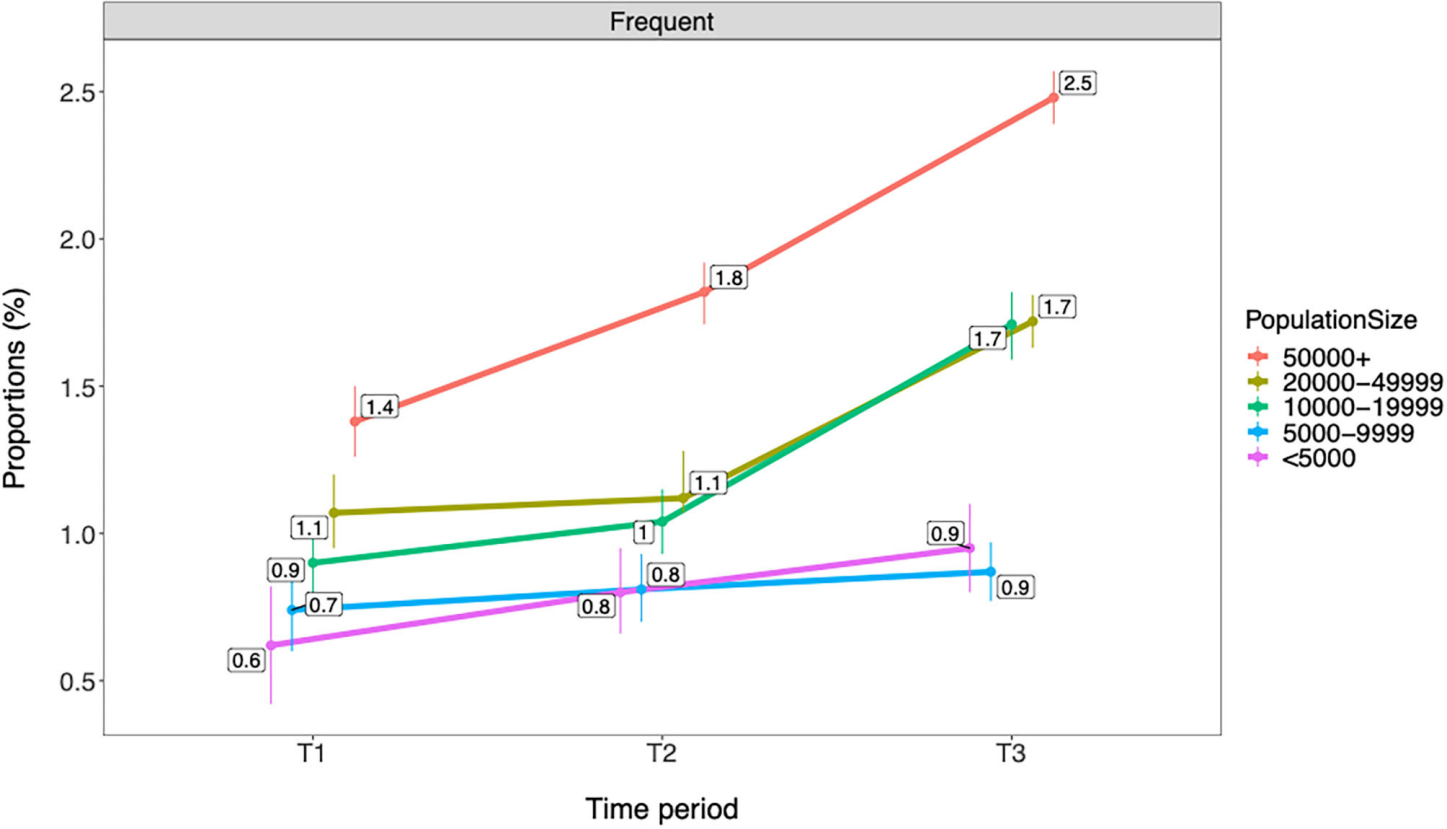
Trends in frequent past year cannabis use by municipality population (*n* = 566,912). T1 = 2010–2013. T2 = 2014–2016. T3 = 2017–2019. Proportions are adjusted for gender and age group/grade. Individuals with missing data on municipality population (*n* = 1,604) are excluded from the analysis. Frequent = frequent past year cannabis use.

Figures 5, 6 show that rates of any and frequent past year cannabis use among Norwegian adolescents were overall higher in municipalities with a larger population. There was a graded difference between the largest municipalities (i.e., +50,000 inhabitants), medium-sized municipalities (i.e., 10,000–49,999 inhabitants), and small municipalities (i.e., <10,000 inhabitants). In the largest municipalities, rates were 9.9% for any and 2.5% for frequent cannabis use compared with 3.9–4.5% for any and 0.9% for frequent cannabis use in the small municipalities. A gradual increase in any and frequent cannabis use was observed from T1 to T3 in the largest municipalities. An increase from T2 to T3 was observed in the medium-sized municipalities for both any and frequent cannabis use and for any past year cannabis use in the smallest municipalities. All changes described above refer to ps ≤ 0.01. See Table 6 for details.

**Table 6:** Trends in any and frequent past year cannabis use by municipality population (*n* = 566,912).

	**(T1)**	**(T2)**		**(T3)**	
	**Prop (95% CI)**	**Prop (95% CI)**	**Change^**a**^**	**Prop (95% CI)**	**Change^**a**^**
**Any use**					
<5,000	2.7 (2.3, 3.1)	3.3 (3.0, 3.6)	–	3.9 (3.6, 4.2)	> T1, T2*
5,000–9,999	3.8 (3.5, 4.1)	3.7 (3.4, 3.9)	–	4.5 (4.3, 4.7)	> T1, T2
10,000–19,999	4.5 (4.3, 4.8)	4.5 (4.3, 4.7)	–	6.8 (6.6, 7.0)	> T1, T2
20,000–49,999	5.2 (4.9, 5.4)	5.1 (4.9, 5.4)	–	6.9 (6.8, 7.1)	> T1, T2
50,000+	6.8 (6.5, 7.0)	8.0 (7.8, 8.2)	> T1	9.9 (9.7, 10.1)	> T1, T2
**Frequent use**					
<5,000	0.6 (0.4, 0.8)	0.8 (0.7, 1.0)	–	0.9 (0.8, 1.1)	–
5,000–9,999	0.7 (0.6, 0.9)	0.8 (0.7, 0.9)	–	0.9 (0.8, 1.0)	–
10,000–19,999	0.9 (0.8, 1.0)	1.0 (0.9, 1.1)	–	1.7 (1.6, 1.8)	> T1, T2
20,000–49,999	1.1 (1.0, 1.2)	1.1 (1.1, 1.3)	–	1.7 (1.6, 1.8)	> T1, T2
50,000+	1.4 (1.3, 1.5)	1.8 (1.7, 1.9)	> T1	2.5 (2.4, 2.6)	> T1, T2

*T1 = 2010/2013; T2 = 2014/2016; T3 = 2017/2019. Prop, proportions; CI, confidence interval*.

a *“>” implies significant increase; “ < ” implies significant decrease; and “–” implies no change compared with previous time period(s)*.

*All changes p < 0.001 unless specified by asterisk: p ≤ 0.01*.

#### Sensitivity Analyses

**Figure 7:**
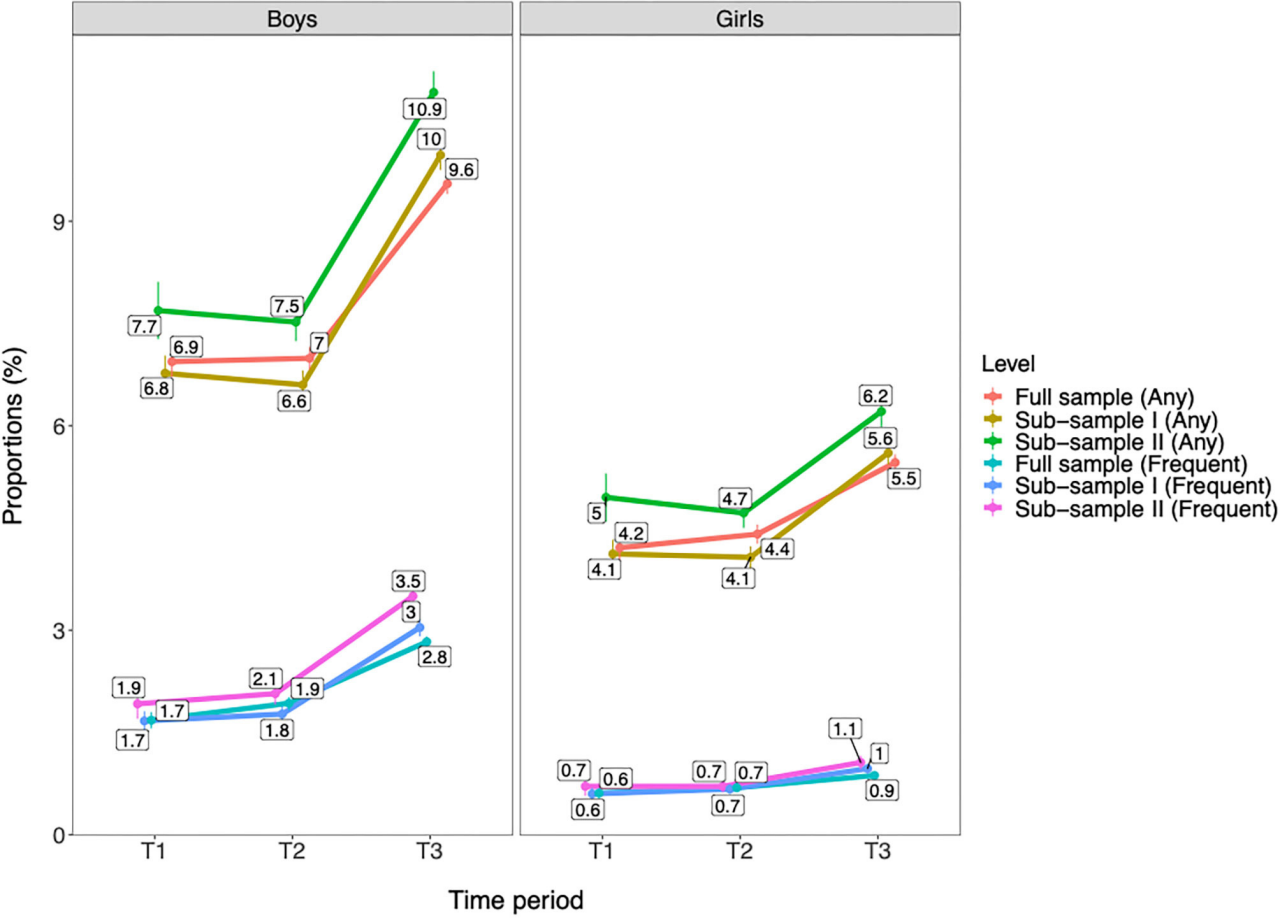
Trends in past year cannabis use by gender across the full included sample and two subsamples with participation in all time periods of T1, T2, and T3. T1 = 2010–2013. T2 = 2014–2016. T3 = 2017–2019. Full sample = the included sample in the present study (Boys: *n* = 280,243; Girls: *n* = 286,669; Total: *n* = 566,912). Subsample I = the sample with exclusively survey responses from municipalities that have participated in T1, T2, and T3 (Boys: *n* = 153,830; Girls: *n* = 157,944; Total: *n* = 311,774). Subsample II = the sample with exclusively survey responses from municipalities that have participated in T1, T2, and T3 and with only one occurrence of participation per time period (Boys: *n* = 86,792; Girls: *n* = 89,787; Total: *n* = 176,579). Any = any past year cannabis use. Frequent = frequent past year cannabis use. Proportions are adjusted for age group, geographical location, and municipality population. Individuals with missing data on municipality population are excluded from this analysis (*n* = 1,604). All changes from T1/T2 to T3 are significant at *p* < 0.001.

Sensitivity analyses were conducted with two subsamples composed of survey responses from municipalities that had participated in all time periods of T1, T2, and T3. The first of these subsamples had any participation in all these time periods (i.e., subsample I; survey responses: *n* = 311,774; unique municipalities: *n* = 144). The second of these subsamples had only one occurrence of participation per time period (i.e., subsample II; *n* = 176,579; unique municipalities: *n* = 85). Trend slopes for past year cannabis use from T1 to T3 was described for these subsamples in comparison with the full included sample (*n* = 566,912). Rates were adjusted for geographical location and municipality population. These analyses showed very similar trend slopes across the three samples for both any and frequent past year cannabis use (Figure 7). Specifically, no significant changes from T1 to T2 were observed, but a considerable increase from T2 to T3 (all ps < 0.001) on both any and frequent past year cannabis use for both genders was confirmed in both subsamples.

## Discussion

The present study indicates that the prevalence of cannabis use has increased from 2014–2016 (T2) to 2017–2019 (T3), but not from T1 to T2, among Norwegian adolescents. With few exceptions, this increase was evident across genders, age groups, geographical locations, and municipality populations. In several of the analyses, a more gradual increase in cannabis use was also observed from T1 (2010–2013) to T3. This was the case for Eastern Norway, the largest municipalities, and to some extent in elementary school-age adolescents. Although fluctuations in rates were observed from T1 to T2 for a specific geographical region (i.e., Western Norway), our findings suggest that cannabis use has become more widespread and normalized among Norwegian adolescents in recent years.

Consistent with previous research (19), boys had a clearly higher cannabis use compared with girls. This pattern was consistent across frequencies of cannabis use (i.e., from only using cannabis once to frequent use) and across age groups. While the boys:girls gender ratio for any past year cannabis use was close to 2:1, it was even larger for frequent cannabis use at ~3:1. Older adolescents had consistently higher past year cannabis use than younger adolescents. This finding is also in line with previous research that suggests a gradual increase in the initiation of cannabis use throughout adolescence, with its peak by early adulthood (18, 28). Importantly, past year cannabis use increased from T2 to T3 for both genders and all age groups. The most notable exception was girls in third upper secondary school, where no distinct pattern of change was observed for either any or frequent use. However, low participation rates in the first Ungdata surveys among upper secondary school-age adolescents limit the validity of the estimates on cannabis use at T1 for this age group. This should be taken into account when interpreting the results.

Adolescents in Eastern Norway reported significantly higher past year cannabis use compared with the rest of the country. This finding is in accordance with the results from recent studies on both adolescents and higher education students, where the Oslo region (the capital of Norway, located in Eastern Norway) had the highest illicit drug use (16, 18). It is difficult to provide a firm conclusion on potential explanations for these geographical differences in cannabis use. There are no formal differences across Norwegian geographical areas in regard to legislation of cannabis use, but it is likely that attitudes toward cannabis use may be more liberal in urban areas (16), which might explain this finding. The present study however did not investigate potential mechanisms for these geographical differences in cannabis use rates, and these are therefore mere speculations. Apart from Eastern Norway, the present study found only very modest differences in past year cannabis use between regions in Norway regarding both any and frequent use. While fluctuation of rates of any past year cannabis use was observed in Western Norway, proportions of past year cannabis use increased across all geographical locations from T2 to T3. Larger municipality population was further associated with higher past year cannabis use, a pattern that was consistent throughout the time periods and across any and frequent use. Interestingly, these differences were graded, indicating that small municipalities had the lowest cannabis use, medium-sized municipalities had higher cannabis use, and the largest municipalities had the highest cannabis use among Norwegian adolescents. These findings support previous studies that indicate higher substance use in urban vs. rural areas (29, 30).

The most pronounced increase in past year cannabis use was observed from T2 to T3, while changes before this time period were more modest in most analyses. These findings may suggest that the observed increase in cannabis use to a large extent represents a very recent change. These findings support very similar results in other studies of Norwegian adolescents and young adults (16, 18). However, several exceptions to this generalization were observed. Importantly, a relatively steep gradual increase in cannabis use was seen from T1 to T3 in Eastern Norway as well as in the largest municipalities (i.e., 50,000 inhabitants or more). In previous research, increased rates of cannabis use is suggested to indicate normalization (14), which is characterized by cannabis being perceived by young people as a substance that is part of the range of drugs typical for nightlife (13), something that is likely to be part of the explanation for our results. This is particularly interesting in relation to the areas with the most gradual increase over time, where the trends are more likely to represent substantial changes in relation to cannabis use. Changes that were only evident from T2 to T3 should, on the other hand, be interpreted with caution. These may either represent temporary fluctuations or be indicating early tendencies toward normalization of cannabis use among Norwegian adolescents. Future follow-up studies of continued trends are needed to disentangle this issue further.

We cannot provide any firm conclusions on the reasons for the observed increase in cannabis use in our study. Several potential mechanisms for changing trends in cannabis use have been proposed, such as increased availability, decreased harm perception, and more liberal attitudes toward use (31). Other individual-level correlates of cannabis use include low self-esteem, conduct problems, and family adversity such as parental mental illness and negative life events (19, 32). Changes within all these domains could potentially contribute to increased rates of cannabis use. Aggregate media coverage is also associated with increased cannabis use (33) and could thus be another factor related to the changing trend among Norwegian adolescents. Potential mechanisms involved in the observed increase of cannabis use should be explored in future research. In this respect, follow-up studies may also be needed to further validate that the observed increase is not related to sampling issues, resulting from different characteristics across the time periods. Although this possibility in part was addressed in the present study, with the comparison of trends between the included sample and the subsamples consisting of only municipalities that participated in all three time periods, further investigation of this issue is encouraged.

Finally, the present study found similar trend slopes for any and frequent cannabis use, suggesting that the observed change in cannabis use in our sample also included an increase in regular cannabis use. This trend is troubling for several reasons. Epidemiological, clinical, and laboratory studies have established associations between regular cannabis use and a range of adverse outcomes (6–11). Regular use is associated with concentration problems and missing classes (9), lower initiative and persistence (7), and adverse effects on psychosocial development and mental health (11). Thus, cannabis use during the adolescent years is an important public health concern that needs to be given attention (11, 34).

### Strengths and Limitations

A considerable strength of the present study is the very large dataset that included over 600,000 responses from Norwegian adolescents, as well as the repeated measures over three time periods spanning over 10 years with comparable data on cannabis use. The Ungdata survey is carried out at school and is generally considered representative of the Norwegian adolescent population. This is highlighted by the large proportion of municipalities in Norway that have participated in Ungdata. Specifically, 410 out of the 428 municipalities in Norway (comprising ~95% of the total number of municipalities) have participated at least once in the Ungdata survey, and 340 municipalities were eligible for analyses in the present study.

#### The Sample

The main limitation of the present study is that the Ungdata survey was not conducted on regular intervals across the municipalities, meaning that prevalence rates for T1, T2, and T3 are not composed of direct comparable municipalities. In our sample, 340 municipalities had participated in T1, T2, or T3, but only 144 out of these eligible municipalities had participated in all of these time periods. In addition, some municipalities have participated more than once within any given time period. This may potentially have led to some bias in the overall proportions of cannabis use per time period due to overrepresentation of some municipalities. However, sensitivity analyses using a subsample with municipalities that participated in all the time periods of T1, T2, and T3—including a subsample with only one participation per time period—provided support of the validity of our findings. These analyses showed no change in past year cannabis use from T1 to T2, as well as a significant increase in both any and frequent cannabis use from T2 to T3 for both genders with a similar magnitude as in the main sample. In addition, a range of measures was undertaken to limit potential bias caused by sampling issues. We summed each of the 10 survey years into 3-year periods, therefore increasing the number of municipalities represented at each time period. All analyses were adjusted by important variables that varied across time periods. We also provided several stratifications by which we assessed trends in cannabis use (e.g., gender, age groups, geographical location, and municipality population), and these different perspectives suggested to a large degree similarity in trends. Thus, our evaluation is that the Ungdata dataset provides a good basis for estimating overall trends in the population.

#### Missing Data

~10% of the total sample had missing data on cannabis use, gender, or age group and were excluded from the analyses. Missing data could potentially lead to some degree of selection bias. Unfortunately, municipalities with very small populations (i.e., <2,000 inhabitants) were overrepresented among survey responses with missing data. This could be explained by the fact that grade levels were often not asked for at the elementary school level in municipalities with few respondents in order to ensure the confidentiality of the respondents. Thus, the results in regard to the smallest municipalities should be interpreted with some caution. Boys were also somewhat overrepresented among the adolescents with missing data, particularly on missing responses related to cannabis use, something that potentially may have resulted in a slight underestimation of cannabis use among boys. Although it is unlikely that missing data would have seriously affected the findings of the present study, these aspects should be taken into account when interpreting the results.

#### Other Potential Limitations

The Ungdata surveys were administered at school level, and our study lacks data on cannabis use among school non-attending individuals. Thus, although the vast majority of Norwegian adolescents attend school (22), the point prevalence rates of cannabis use in the present study may not be fully generalizable to the total adolescent population. On the other hand, the comparability of prevalence rates across survey years is not affected by this limitation. Cannabis use could potentially be underreported in studies based on self-report due to the stigmatized and undesirable behavior in question. However, convergent validity of self-reported cannabis use and urine tests appears to be satisfactory (35, 36), and self-reported measures are often necessary in large-scale community-based studies. Multiple testing is another potential limitation. Due to a relatively strict criteria for significance (i.e., *p* ≤ 0.01), the likelihood is, however, reduced for detecting false-positive changes across survey years related to multiple testing. On the other hand, we may have underreported some minor fluctuations in proportions of cannabis use across survey years. Finally, we operationalized frequent cannabis use as “11 times or more” during the past year. It may be argued that a higher cutoff would better capture frequent use. However, this cutoff was the highest available in our data.

## Conclusions

Rates of past year cannabis use among Norwegian adolescents appear to have increased significantly from 2014 to 2016 to 2017 to 2019, and for subgroups of adolescents, a gradual increase from 2010 to 2013 was also observed. These changes were to a large extent evident across genders, age groups, geographical locations, and municipality populations, with some exceptions. Our findings may thus suggest that cannabis use has been more widespread and normalized among adolescents in Norway during the most recent years. Preventive interventions to hinder initiation of cannabis use as well as measures to address regular cannabis use among Norwegian adolescents are warranted.

## Data Availability Statement

Publicly available datasets were analyzed in this study. This data can be found here: https://ungdata.no. Data and materials in the Ungdata-surveys are included in a national database administered by Norwegian Social Research (NOVA). Data are available for research upon application.

## Ethics Statement

The studies involving human participants were reviewed and approved by Norwegian Centre for Research Data (NSD). Written informed consent to participate in this study was provided by the participants' legal guardian/next of kin.

## Author Contributions

OH wrote the manuscript, prepared the dataset, planned the data analyses, and conducted the data analyses. SN, KS, and KB contributed to the planning of the data analyses, and SN also contributed to the conduct of data analyses. KS applied for and prepared the data set. All authors were involved in the interpretation of the results and critically reviewed the manuscript, and read and approved the final manuscript.

## Conflict of Interest

The authors declare that the research was conducted in the absence of any commercial or financial relationships that could be construed as a potential conflict of interest.
