# Supplementary material for: Past Year Cannabis Use Among Norwegian Adolescents: Time Trends Based on the Ungdata Surveys 2010–2019
Source: Front Psychiatry. 2021 Mar 16;12:627479. doi: 10.3389/fpsyt.2021.627479 (PMC8007792; doi:10.3389/fpsyt.2021.627479)
Supplement: Supplementary file 1 [file Table_1.DOCX]

*Appendix 1. Operationalization of the geographical location variable (n=628,678)*

| **Variable value** | **Counties ^1^** | **Municipality and county codes in Ungdata** | **Frequency** | |
| --- | --- | --- | --- | --- |
| Geographical location | Name | Range | Total (n) | Missing municipality code (n) |
| “Eastern Norway” | Østfold | 101-138 | 39,706 | 0 |
|  | Akershus ^2^ | 211-239 | 94,387 | 0 |
|  | Oslo | 301 | 57,982 | 0 |
|  | Hedmark | 402-441 | 20,132 | 0 |
|  | Oppland | 501-545 | 23,824 | 0 |
|  | Buskerud | 600-633 | 27,189 | 17 |
|  | Vestfold | 701-728 | 21,439 | 0 |
|  | Telemark | 800-834 | 23,772 | 427 |
| “Southern Norway” | Aust-Agder | 900-941 | 17,686 | 549 |
|  | Vest-Agder | 1000-1046 | 27,039 | 236 |
| “Western Norway” | Rogaland | 1101-1160 | 82,831 | 0 |
|  | Hordaland | 1201-1266 | 43,841 | 0 |
|  | Sogn og Fjordane ^3^ | 1400-1449 | 8,851 | 15 |
|  | Møre og Romsdal | 1502-1576 | 26,613 | 0 |
| “Central Norway” | Sør-Trøndelag | 1601-1665 | 32,253 | 0 |
|  | Nord-Trøndelag | 1702-1756 | 21,032 | 0 |
| “Northern Norway” | Nordland | 1800-1874 | 35,687 | 183 |
|  | Troms ^4^ | 1900-1943 | 15,691 | 218 |
|  | Finnmark | 2000-2030 | 8,452 | 177 |
|  | Svalbard | 2100 | 271 | 0 |
| Total |  |  | 628,678 | 1,822 |
|  |  |  |  |  |
| ^1^ By organization 1^st^ of January 2019. | | | | |
| ^2^ One municipality code was recoded from 100217 to 217. | | | | |
| ^3^ One municipality code was recoded from 101400 to 1400. | | | | |
| ^4^ One municipality code was recoded from 5421 to 1900. | | | | |
