# Supplementary material for: Past Year Cannabis Use Among Norwegian Adolescents: Time Trends Based on the Ungdata Surveys 2010–2019
Source: Front Psychiatry. 2021 Mar 16;12:627479. doi: 10.3389/fpsyt.2021.627479 (PMC8007792; doi:10.3389/fpsyt.2021.627479)
Supplement: Supplementary file 2 [file Table_2.DOCX]

*Appendix 2. Missing data in the full Ungdata-sample (n=628,678)^1^*

|  | **Valid responses**, n = 566,912*^1^* | **Missing gender**, n = 17,442*^1^* | **Missing age**,  n = 20,063*^1^* | **Missing cannabis**,  n = 20,174*^1^* | **Missing multiple**,  n = 4,087*^1^* |
| --- | --- | --- | --- | --- | --- |
| **Gender** | | | | | |
| Boys | 280,243 (49.4%) | 0 | 10,431 (52.0%) | 13,490 (66.9%) | 631 (63.9%) |
| Girls | 286,669 (50.6%) | 0 | 9,632 (48.0%) | 6,684 (33.1%) | 356 (36.1%) |
| Unknown | 0 | 17,442 | 0 | 0 | 3,1 |
| **Grade** | | | | | |
| Elementory 8th | 119,574 (21.1%) | 3,158 (18.1%) | 0 | 3,162 (15.7%) | 135 (12.6%) |
| Elementory 9th | 116,658 (20.6%) | 3,383 (19.4%) | 0 | 3,130 (15.5%) | 168 (15.7%) |
| Elementory 10th | 117,813 (20.8%) | 3,953 (22.7%) | 0 | 3,255 (16.1%) | 178 (16.6%) |
| Upper 1st | 103,679 (18.3%) | 3,322 (19.0%) | 0 | 5,005 (24.8%) | 296 (27.6%) |
| Upper 2nd | 69,432 (12.2%) | 2,215 (12.7%) | 0 | 3,645 (18.1%) | 203 (18.9%) |
| Upper 3rd | 39,756 (7.0%) | 1,411 (8.1%) | 0 | 1,977 (9.8%) | 93 (8.7%) |
| Unknown | 0 | 0 | 20,063 | 0 | 3,014 |
| **Geographical location** | | | | | |
| Eastern Norway | 281,199 (49.7%) | 7,946 (45.7%) | 5,476 (27.3%) | 10,978 (54.5%) | 1,953 (47.9%) |
| Western Norway | 146,245 (25.8%) | 5,608 (32.3%) | 5,034 (25.1%) | 4,230 (21.0%) | 1,004 (24.6%) |
| Northern Norway | 52,354 (9.2%) | 1,354 (7.8%) | 3,859 (19.2%) | 2,098 (10.4%) | 436 (10.7%) |
| Mid-Norway | 46,244 (8.2%) | 1,780 (10.2%) | 3,420 (17.1%) | 1,380 (6.8%) | 461 (11.3%) |
| Southern Norway | 40,074 (7.1%) | 698 (4.0%) | 2,260 (11.3%) | 1,466 (7.3%) | 227 (5.6%) |
| Unknown | 796 | 56 | 14 | 22 | 6 |
| **Municipality size** | | | | | |
| <2000 | 1,648 (0.3%) | 81 (0.5%) | 6,621 (33.0%) | 45 (0.2%) | 418 (10.3%) |
| 2000-4999 | 35,142 (6.2%) | 1,006 (5.8%) | 4,489 (22.4%) | 1,003 (5.0%) | 384 (9.4%) |
| 5000-9999 | 69,268 (12.3%) | 2,167 (12.5%) | 1,960 (9.8%) | 2,313 (11.5%) | 437 (10.7%) |
| 10000-19999 | 105,023 (18.6%) | 3,152 (18.2%) | 2,008 (10.0%) | 3,620 (18.0%) | 1,154 (28.4%) |
| 20000-49999 | 143,172 (25.4%) | 4,817 (27.8%) | 3,994 (19.9%) | 4,698 (23.4%) | 706 (17.4%) |
| 50000+ | 210,259 (37.2%) | 6,080 (35.1%) | 967 (4.8%) | 8,378 (41.8%) | 967 (23.8%) |
| Unknown | 2,4 | 139 | 24 | 117 | 21 |
| **Past year annabis use** | | | | | |
| Any use | 37,519 (6.6%) | 1,241 (7.1%) | 744 (3.7%) | 0 | 181 (10.7%) |
| Unknown | 0 | 0 | 0 | 20,174 | 2,398 |
| **Time period** | | | | | |
| T1 (2010-2013) | 121,767 (21.5) | 5,316 (3.8) | 10,802 (7.6) | 2,230 (1.6) | 1,439 (1.0) |
| T2 (2014-2016) | 172,877 (30.5) | 4,767 (2.5) | 3,424 (1.8) | 7,829 (4.1) | 1,125 (0.6) |
| T3 (2017-2019) | 272,268 (48.0) | 7,359 (2.5) | 5,837 (2.0) | 10,115 (3.4) | 1,523 (0.5) |
|  |  |  |  |  |  |
| *^1^*Statistics presented: n (%) | |  |  |  |  |
